# Is the healthcare provision in the Netherlands compliant with universal health coverage based on the right to health? A narrative literature review

**DOI:** 10.1186/s12992-022-00831-7

**Published:** 2022-04-02

**Authors:** Thomas Kuipers, Remco van de Pas, Anja Krumeich

**Affiliations:** 1 National Institute of Public Health and Environment, Antonie van Leeuwenhoeklaan 9, 3721 MA Bilthoven, The Netherlands; 2 Department of Public Health, Institute of Tropical Medicine, Nationalestraat 155, 2000 Antwerp, Belgium; 3 Department of Health, Ethics, and Society, Maastricht University, Minderbroedersberg 4-6, 6211 LK Maastricht, The Netherlands

**Keywords:** Universal health coverage, Right to health, The Netherlands, Health equity, Health system financing

## Abstract

Universal health coverage, as one of the targets of the Sustainable Development Goals, is the access to key promotive, preventive, curative and rehabilitative health interventions for all at an affordable cost. It is a practical expression of the concern for health equity and the right to health, and a goal for all countries. This review is a novel attempt to explore the healthcare provision in the Netherlands as an expression of universal health coverage based on the right to health.

The study adopted a narrative review approach using a framework that consists of 10 universal health coverage indicators which are derived from seven human rights principles. The techno-economic approach to healthcare provision by the Dutch state achieves a healthcare system where most of the population is covered for most of the services for most of the costs. The Dutch state complies with its minimum core obligations, while less attention is paid to participatory decision making and non-discrimination principles. However, with the fiscal sustainability of healthcare provision showing erosion, basing healthcare policy on values based on human rights principles might prevent a regressive policy.

## Background

### Universal health coverage

Inequalities in levels of income, opportunities and health outcomes remain a cause for concern in societies, including high-income countries (HICs), and even more so in light of the COVID-19 pandemic [1–3]. All countries have a moral obligation to address fairness and social justice in the distribution of collective goods like healthcare. Attempts within public health to properly address the societal factors that are relevant to complex health problems have been insufficient, which is partly exacerbated by the absence of a coherent conceptual framework [4]. One such framework is based on human rights, which have gained attention in global development policies such as the Sustainable Development Goals (SDGs). It has also gained attention in regional and national efforts to address inadequate access to healthcare [5].

In this context, the World Health Organization (WHO) proposed Universal Health Coverage (UHC) as a target for the health-related goal on the 2030 Agenda for Sustainable Development, which was unanimously adopted during the 2015 United Nations General Assembly [6, 7]. The 2005 World Health Assembly (WHA) resolution on *Sustainable health financing, Universal coverage and social health insurance* defined UHC as the “*access to key promotive, preventive, curative and rehabilitative health interventions for all at an affordable cost, thereby achieving equity in access*” [8]. The 2010 World Health report on *Health system financing: the path towards universal health coverage* elaborated as to how countries can modify their health systems to achieve UHC by identifying the three UHC dimensions: *what*, *who* and the *proportion of costs* that is covered [9]. These dimensions concern the extent of services included, the population that is covered for these services, and the proportion of the costs that is covered by pooled funds versus out-of-pocket payments (OOPPs). OOPPs are direct costs that can result in financial hardship or even ‘catastrophic health expenditure’, the inability to pay for healthcare due to high costs in relation to income [9].

### The right to health and health equity

In addition to these technical UHC dimensions, the WHO’s normative conceptualization indicates UHC to be *“by definition, a practical expression of the concern for* *health equity and the right to health”* [6]. Health equity and the right to health are terms that should be handled with care though, as their interpretations and implications are contended among scholars and subjected to debate. Firstly, *health equity* is a concept related to justice and qualifies some *inequalities* as *inequities* [10]*. Health inequities* are defined by “*differences which are unnecessary and avoidable but, in addition, are also considered unfair and unjust”* [11]*.* These inequities are at the conceptual foundation of the right to health, and provide moral principles to resolve conflicts between human rights and to locate rights in relation to other social values and goals [12, 13].

Secondly, the legal conceptualization of the right to health underpinning this review is based on the formulation in the 1966 International Covenant on Economic, Social, and Cultural Rights (ICESCR). General Comment 14, an explanatory document issued by the Committee on Economic, Social and Cultural Rights (CESCR), states that “*the right to health must be understood as a right to the enjoyment of a variety of facilities, goods, services and conditions necessary for the realization of the highest attainable standard of health”* [14]*.* Furthermore, it normatively prescribes that health facilities, goods and services should be *available*, *accessible*, *acceptable*, and of good *quality*. UHC is intended to address these conditions of healthcare, generally referred to as the AAAQ framework, to advance the right to health [15]. It takes into account the limited resources of states, irrespective of their wealth, and requires states to *take steps* towards the realization of the right to health in a *progressive* manner [16].

To integrate the concepts of the right to health and UHC, Ooms et al*.* have identified seven important human rights principles that the Committee applies in their elaboration of the right to health in General Comment 14 [10]. The right to health demands states to abide by the principles of *minimum core obligations*, *progressive realization*, *non-discrimination*, *cost-effectiveness*, *participatory decision making*, *shared responsibility*, and the *attention to vulnerable or marginalized groups*. Table 1 portrays a synthesis of these seven human rights principles and the UHC components as proposed by Sridhar et al*..* The result is a normative framework, consisting of 10 measurable and achievable indicators, that evaluates the extent to which UHC is based on the right to health (see Table 1) [17].

**Table 1:** Ten indicators for UHC based on the Right to Health [17]

Indicator	Underlying legal principle
1. Existence of a legal mandate for UHC in the country	Minimum core obligation / progressive realization
2. Extent of coverage in terms of depth (which services are covered)	Minimum core obligation / progressive realization
3. Extent of coverage in terms of breadth with attention to equity (who is insured)	Minimum core obligation / progressive realization
4. Extent of coverage in terms of height with focus on reduction in share of OOPP for health care (what proportion of costs are covered)	Minimum core obligation / progressive realization
5. Commitment of adequate resources to deliver UHC with focus on percentage of gross national product for healthcare	Minimum core obligation / progressive realization
6. Cost-effectiveness with attention to equity	Cost-effectiveness / nondiscrimination
7. International assistance as a percentage of GDP	Shared responsibility
8. Existence of an international development policy explicitly including specific provisions to promote and protect the right to health	Shared responsibility
9. Service Availability and Readiness Assessments (SARA)^a^ on participatory decision making	Participatory decision making / nondiscrimination
10. SARA assessment on prioritization of marginalized groups	Attention to vulnerable and marginalized groups / nondiscrimination

^a^Service Accessibility and Readiness Assessment (SARA), developed by the WHO and USAID, is a health facility assessment tool utilizing a systematic survey to generate reliable and objective data to assess and monitor the accessibility of health services and readiness of the health sector. Available at: https://www.who.int/data/data-collection-tools/service-availability-and-readiness-assessment-(sara)

### The healthcare landscape and key reforms in the Netherlands

Historically, the Dutch healthcare system included a mix of public and private health insurance schemes. By the end of the 1960’s, this formed a compromise between the faith-based socialistic norms of broad accessibility to a public healthcare system and principles rooted in liberalism of a private health system based on conditions set by the government [18]. It consisted of private insurance for the wealthy and social insurance for the rest. However, this two-tiered healthcare system exacerbated health inequalities between socio-economic groups in the Dutch society [19]. In addition, various major issues had been on the healthcare agenda for years: an increased tension between supply of and demand for health care, the need for cost-containment, upholding solidarity principles and the increasing pressure on accessibility [18].

A culmination of decades’ worth of deliberation and discussion to address these ‘maladies’ in the health system led eventually to a major health insurance reform for the curative care sector in 2006 by introducing market principles to the health insurance landscape [19]. Furthermore, a major reform of long-term care starting in 2015 due to pressing budget deficits after the financial crisis and an increasing demand for an ageing population. In order to improve efficiency and affordability, long-term care was decentralized to the municipalities or dispersed to health insurers, while care at home and self-reliance was promoted. Seemingly, municipalities are in a better position to more efficiently tailor care to the citizens’ needs. Table 2 outlines the current health finance and policy landscape for the various healthcare sectors in the Netherlands.

**Table 2:** The healthcare provision policy landscape of the Netherlands [20]

*Curative care*
The 2006 Health Insurance Act (Zorgverzekeringswet, Zvw) introduced managed competition to the health insurance sector**.** It aimed to reduce central governance, promote efficiency, and improves access at acceptable societal costs [21]. The reform gave citizens more financial responsibilities, and more influence and choice over their healthcare plan. All inhabitants of the Netherlands are by law obligated to participate in the insurance scheme [20]. The government adopted a more distant supervisory role with the 2006 Health Care Market Regulation Act (Wet Marktordening gezondheidszorg, Wmg) and remained responsible at national level for the accessibility, affordability and quality of care [21]. Although the insurance scheme is executed by private firms, its regulation is based on solidarity principles and can in essence be characterized as social [20]. The rational for introducing the competition was to improve quality and affordability of healthcare. Subsequently, three healthcare markets emerged between the insured people, healthcare providers and healthcare insurers [21]. The first one is the health insurance market, where citizens purchase a health insurance plan from one of the private health insurers. The second is the healthcare purchasing market, where insurers purchase care for insured population from the healthcare providers. Lastly, on the healthcare provision market patients utilize healthcare from the healthcare providers, although insurers may impose restrictions on the choice of providers in return for a discount on the insurance plan.
*Long-term care and decentralized care*
There are many services not covered by an insurance scheme under the Health Insurance Act. These services are financed through various other mechanisms and their legal basis are dispersed over multiple acts. The 1968 Exceptional Medical Expenses Act (Algemene wet bijzondere ziektekosten, Awbz) covered the high costs of nursing, treatment and personal care that was not part of the health insurance. It was abolished in 2015 followed by major reforms in the fields of long-term care, social support and youth care [21]. The Long-Term Care Act (Wet Langdurige Zorg, Wlz) provides institutional care for all citizens who need around the clock supervision, which can either be provided at home or in a residential long-term care facility [22]. The institutional care is financed through a general fund that consists of contributions from the state budget and income dependent cost-sharing. The Social Support Act (Wet Maatschappelijke Ondersteuning, Wmo) provides help for domestic care or social support through a decentral and provision-based approach. Its objective is for municipalities to support citizens to participate in society. Municipalities are free to tailor the support for their citizens, and professional care can be substituted with other care solutions, for example care provided by volunteers, neighbors or family. Under the Youth Act (Jeugdwet), care is provided to all children under the age of 18, and their parents if parenting problems and mental problems are indicated [23]. Under the Public Health Act (Wet Publieke gezondheid, WPg), the municipalities are also responsible for services related to disease prevention, health promotion and health protection [21].

### Universal health coverage in the Netherlands

It remains unclear to what extent healthcare provision in the Netherlands is compliant with UHC as an expression of the right to health. Although the *universality* in UHC ostensibly suggests that everyone should be covered, not a single country has achieved complete coverage. This provides the opportunity for all to make progress [24–26]. While the international community identified UHC as the way forward in strengthening resilient health systems, the discussion on the relevance of UHC seems to focus on low- and middle-income countries rather than HIC’s [24]. This is in part due to a common notion suggesting that many HIC’s have already fulfilled the targets of UHC. Also, the absence of a relevant UHC monitoring framework applicable to HIC’s plays a role, as Bergen et al. argues [24]. HIC’s like the Netherlands might be expected to meet higher standards in fulfilling the right to health when compared to low- and middle-income countries.

This review is therefore a novel attempt to relate UHC to a HIC such as the Netherlands, with the ‘UHC based on the Right to Health’ framework (see Table 1) as a lens to analyze the Dutch policy landscape. We acknowledge that the implementation of the right to health goes beyond the implementation of UHC, also in the Netherlands. It includes, amongst others, action on the Social Determinants of Health and should address the health(care) needs of specific marginalized groups, such as undocumented migrants. For this review, we follow Ooms et al. analyses that, in the context of the Sustainable Development agenda, UHC can be considered a practical expression of the right to health *care*, in essence a subset of the broader Right to Health concept. We explore the health service provision as an expression of UHC based on the right to health, and aim to contribute to the debate on UHC in HIC’s and the use of rights-based approaches to health systems’ financing and organization.

## Methodology

### Research design

The study adopted a narrative literature review design. A narrative review is a type of non-systematic literature review that is used to for more general debates and can serve to provoke thought and dialectic exchange since it combines theory and context [27]. This review followed a methodological approach described in previous publications, which includes: a preliminary literature search, a description and synthesis of the available topical literature, and reports it using the necessary elements of a narrative review [27–29]. Predefined concepts are tested through the collection, aggregation, and triangulation of empirical data to synthesize empirical statements [30]. The review entails a narrative synthesis of previously published information on the subjects of UHC and the right to health in the Netherlands. Its purpose is to provide empirical and broader perspective on these subjects, and hence not to provide a comprehensive systemic review.

### Data collection

Data was collected through the use of PubMed and Google Scholar or purposively collected from various (inter)governmental organizations. The data that this review utilizes is collected from both qualitative and quantitative academic research (*n* = 31) and grey literature (*n* = 42) sources, among which from governmental organizations (*n* = 8), non-governmental organizations (*n* = 20) and intergovernmental organizations (*n* = 14). It includes various types of data such as academic research, policy briefs, reports, book chapters, websites, health information databases, legal documents and acts, and epidemiological and public health documentation. A specific search for case studies, including academic, legal and grey literature reporting on human rights violations in relation to health care provision in the Netherlands, complemented the data collection.

The data was purposively searched using key terms based on each of the frameworks indicators. The data was assessed for various inclusion criteria; accessibility in the public domain, either in print or digitally, written in English or Dutch, and the most recent documentation providing it to be no earlier than 2004. The data utilized is from the public domain, in print and accessible on the web or in a library. Through an iterative process, the executive summaries and abstracts of the literature were reviewed and critically appraised, and the literature relevant to the indicator was subsequently included until enough data saturation occurred to inform the indicator.

### Theoretical framework

The ‘UHC based on the Right to Health’ framework (see Table 1) allows for a concise synthesis for each of the 10 indicators. Sridhar et al. described three major challenges with indicator development: data availability, universality of targets, and the adaptability of global goals to local populations. These indicators have been proposed to measure the achievement of UHC that captures the right to health principles in a measurable, achievable and sustainable manner [17].

The indicators from the right to health framework might not capture the priorities of the Netherlands. It should be recognized that the process of determining these targets involve political considerations. Therefore, while interpreting the results, emphasis should be placed on the right to health principles behind each indicator, rather than whether or not the Netherlands is explicitly compliant with each indicator. Also, the definition of UHC varies among experts. The WHO’s conceptualization of UHC is used in this review, as it is considered the international mandated authority on health. The covenant and the general comment 14 serves as the legal international framework on the definition of the right to health and its interpretation.

Furthermore, the right to health is broader than just health care and includes other determinants of health. The 10 indicators of the UHC framework based on in the right to health are about health*care* rather than health, and do not include other determinants of health. In the context of the Sustainable Development Goals, Sridhar et al*.* have proposed these 10 indicators to capture the achievement of the principles that flow from the right to health. yet can also be operationalized to generate measurable, achievable, sustainable indicators. The implementation of the SDG’s is a global responsibility, and is required to be likewise implemented in, and periodically reviewed for, high-income countries, including the Netherlands [31]. Given these global sustainable development requirements, this review focused on analyzing the right to healthcare using the 10 indicators proposed by Sridhar et al. [10].

## Results

### UHC based on the right to health

The first five indicators of the framework deal with the minimum core obligations of a state and whether or not these obligations are realized in a progressive manner. The Netherlands has an implicit legal mandate for UHC (*Indicator 1*) which consists of a vast variety of different acts and legislation within national, regional and global jurisdictions.

On an international level, the Netherlands is a signatory to the Universal Declaration of Human Rights and the United Nations Principles for Older Persons, and has also ratified the ICESCR and the Convention on the Rights of Persons with Disabilities [32]. The UHC mandate on a regional level is derived from the European Convention of Human Rights, the European Social Charter, and the Convention for the Protection of Human Rights and Dignity of the Human Being with regard to the Application of Biology and Medicine [32]. On a national level, the following five acts form the legal basis for healthcare provision: the Health Insurance Act for curative healthcare services, the Long-term Care Act for institutional care, the Social Support Act for domestic care and social support, the Youth Act for care for citizens under 18 years of age, and the Public Health Act for disease prevention, health promotion, and health protection (see Table 2) [21]. More than dozens of other domestic laws arrange how healthcare and its financing is shaped, including both legislation specific to health and more general legislation concerning health.

The next indicators relate to the three dimensions of UHC; *what*, *who* and the *proportion of costs* that are covered. The benefit package for basic health insurance under the 2006 Health Insurance Act covers a broad set of healthcare services (*Indicator 2*), including but not limited to: care provided by hospitals, general practitioners, and medical specialists, midwifery care and maternity care assistance, medical aids and devices, transportation, pharmaceutical care, and primary and secondary ambulatory mental health care [33]. Most non-essential healthcare services (routine dentalcare, physiotherapy, complementary and alternative medicines etc.) and elective procedures are either partially covered or excluded from the basic health insurance coverage and included in supplementary insurance packages instead. Domestic care, youth care, prevention services and social support is founded in separate legal acts. These services are delivered on a decentral level by the municipalities and funded by the state budget and income dependent cost-sharing [21].

All residents in the Netherlands and Dutch citizens abroad who pay income tax to the Dutch state are obligated under the Health Insurance Act to be insured for this basic health benefit plan (*Indicator 3*). Parallel health systems or arrangements exist for military personal, undocumented migrants and people that refuse to be insured based on religious beliefs or certain world views [21]. A small minority of the population (1.31%) is reported to be either uninsured or defaulting on their payments [34].

The Netherlands operates a complex cost-sharing system including mandatory and voluntary deductibles (a form of out-of-pocket payment that is paid per year before insurance covers the remaining expenses), own contributions, insurance premiums, and income taxation (*Indicator 4*) [21]. The mandatory deductible is levied on all healthcare expenses with the exception of general practitioner consultations, maternity care, home nursing care, and integrated care. An additional voluntary deductible can by chosen by the insured which results in a discount on the insurance premium. The OOPPs have increased in the recent years, mainly due to an increase in the mandatory deductible and a cost shift from public to private sources by excluding services from the basic benefit package [21].

The Netherlands is among the world’s biggest healthcare spenders. Its health expenditure has risen considerably in the last decades due to greater propensity to use tertiary care, the introduction of new technologies, and the relaxation of fiscal restrictions on health expenditure (*Indicator 5*) [35, 36]. The majority of the expenditure is concentrated on the chronically ill, the elderly, and the dying.

Due to the rising healthcare costs in the Netherlands, there is much attention to the cost-effectiveness of healthcare interventions. While cost-effectiveness is an important principle of the insurance package, in practice it plays a substantial role mainly in the assessment of pharmaceuticals [37, 38]. Cost-effectiveness is addressed by many research programs and initiatives invested in efficiency research, as well as by improving the use of cost-efficiency in healthcare by legal and economic incentives [21, 39–41].

The 7^th^ and 8^th^ indicators concern the shared responsibility for UHC, as states and other actors that are in a position to assist must indeed do so to a minimum threshold [17]. They capture how much a state spend on official development assistance (ODA) and whether or not the right to health is stated in their foreign development policy. Due to shifting priorities, the Dutch government has not met the internationally accepted target of spending 0.7% of GDP on official development assistance in recent years (*Indicator 7*) [42]. Nonetheless, health is a prominent area for the Dutch ODA with 8.5% of the ODA budget spend on Global health in 2015 [43]. The Netherlands has a long history in protecting and promoting human rights and is party to UN human rights covenants and nearly all human rights conventions. Sexual and reproductive health and rights (SRHR) is the dominant focus of international health policy, used by the Netherlands to invest in health system strengthening. Nevertheless, the Dutch international development policy does not explicitly promote and protect the right to health or UHC (*Indicator 8*) [44–47].

The Netherlands has no SARA assessment on participatory decision making (*Indicator 9*) [48]. Participatory decision making can vary in terms of its deliberativeness, perspective, and decision power. In the last decades, patients have been more involved in the decision-making process, mainly visible in the involvement of patient organizations in hearings, and research and protocol development. The increase of patient involvement has mostly been due to patients’ rights in patient-healthcare provider relationships and the freedom of choice in the healthcare market. Various mechanisms have a legal basis for patient participation on a more institutional and organizational level [49]. For example, patients can seek influence on the (budgeting) policies of long-term care facilities by the means of client councils. Also, health insurers are required under the Health Insurance act to involve patients in the decisions around the healthcare purchases. Nevertheless, public representation is often limited to the non-deliberative consultation of patients. Examples of involvement of citizens, rather than patients, in the decision-making process around the funding of interventions using collective means have been limited [41]. A recent initiative called *Burgerforum, keuzes in de zorg* utilizes a citizens panel to inform the ministry of health on which healthcare services citizens find important to be covered in the basic insurance package [50].

As with the previous indicator, the Netherlands has no SARA assessment on the prioritization of marginalized groups (*Indicator 10*) [48]. Vulnerable and marginalized groups in the Netherlands are heterogeneous groups, among which people with (long term) physical and psychosocial problems, secluded elderly, addicts, homeless people, ethnic minorities, documented and undocumented migrants, and people of lower socioeconomic status. The insurance coverage and its impact can vary for some of these groups.

For example, some key articles indicate that the high-income class spends less than 1% of their income on healthcare in the form of OOPPs, compared to more than 4% for the low-income class [21, 51]. Meanwhile, the difference of the amount of years perceived to be in good health between the lowest and highest income class has been 18.2 years [52]. Inequalities of the quality adjusted-life years expectancy between the low and highly educated have also widened [53].

Also, undocumented migrants and asylum seekers are excluded from health insurance under the Health Insurance Act and covered through parallel arrangements. In principle, undocumented migrants have access to the same healthcare services covered in the basic benefit package and providers are obliged to provide the services. However, OOPPs are required for the care they receive. As this relates to people who are often not able to pay, healthcare providers are partially reimbursed by the government if certain terms are met [21]. Furthermore, asylum seekers are covered for care that is comparable to the basic benefits package. The access to healthcare for asylum seekers is regulated through the Asylum Seekers Care Regulation (*Regeling Zorg Asielzoekers*). Asylum seekers are listed with special asylum seekers health centers and do not have to pay mandatory deductibles or insurance [21].

## Discussion

### Minimum core obligations and progressive realization

The overall health financing and care policy environment in the Netherlands indicates that healthcare coverage is compliant with a conception of UHC based on the right to health. The first proportion of the UHC based on the right to health analysis provides an overall positive impression when it’s evaluated for its compliance with the legal principles as is set out in the ICESCR. The underlying legal principles behind these indicators are the state’s *minimum core obligations* and *progressive realization*. Embedded in a vast body of legislation, most essential and curative healthcare services are covered for most people living in the Netherlands. In other words, a large proportion of the costs are covered for most services for most people. While this might be expected in most HIC’s like the Netherlands, it would be useful to discuss the trends considering the rising healthcare costs. Although essential services are provided and accessible to most people, cost-containment of healthcare expenditure can potentially lead to retrogression in the realization of the right to health.

For example, the effects of the reforms privatizing the health insurance system are to be considered here [49]. In accordance with the 2006 Health Insurance Act, healthcare providers are incentivized to differentiate themselves by price and services via the selective contracting of health insurers [54]. Selective contracting in addition to the quality and quantity requirements can put pressure on the accessibility of care, especially in areas with low population density [49]. For example, multiple hospitals filed for bankruptcy in 2018 due to quality and quantity of care issues [55]. The introduction of market principles to the health insurance sector as well as the decentralization and reallocation of the long-term care (accompanied with large budget cuts), have contributed to slowdown the growth of the total health expenditure [21, 56]. However, it remains to be seen the coming decades if these efforts for cost-containment are sustainable as the population continues to age [21, 36]. Furthermore, trends and projections of health inequities show that they continue to persist or even increase [21]. These health inequalities are linked to societal determinants like income, work, education, urbanization, nationality, lifestyle, healthcare accessibility etc. For example, the adverse health effects due to unemployment after the recent economic recession are becoming apparent and mainly affect people with a lower income or other vulnerable groups [21]. The socio-economic consequences of the COVID-19 pandemic might also further exacerbate health inequalities.

Another example are the various austerity measures that were part of these cost-containment efforts. One of the them relates to the rise of OOPPs due to the gradual shift of public to private sources [21]. This is done by an ongoing limitation of services covered by the Health Insurance Act or the Long-Term Care Act [49]. For instance, various non-essential healthcare services like physiotherapy, routine dentistry care, occupational therapy, exercise therapy and dietary therapy are excluded from the basic insurance package or conditionally included [21]. They are subsequently covered by supplementary insurance plans for which (contrary to the basic insurance package) health insurers are allowed to differentiate the premiums according to a patient’s risk profile. So-called high-risk groups, like the chronically ill, elderly, disabled and psychiatric patients, can count on a higher premium in order to be insured for these services [49]. Furthermore, these services might only be accessible to people who are able to pay for it privately, raising all sorts of concerns regarding equity and the distribution of services.

Another austerity measure has been the gradual increase of the mandatory insurance deductible [49]. The mandatory deductible serves to reduce the moral hazard of being insured, which is the use of more or extra health services just because the expenses are covered by the insurance. It has been gradually raised form €150 (in 2008) to €385 (in 2020) and contributed to the increase of OOPPs [21, 57]. In the Netherlands, OOPPs make up for a relatively small proportion of total spending compared to other OECD countries [36]. However, the insurance deductibles have not been included in the statistics for some reason. Furthermore, the burden of OOPPs are unequally distributed and tilted towards the people of lower socioeconomic status and the so-called high-risk groups. Data from household income and expenditure surveys identified by the WHO suggests that if OOP spending is reduced to levels lower than 15% of total health expenditure, few households would be engaged in catastrophic health expenditure [58]. Thus, while the *minimum core obligations* are largely fulfilled, the principle of *progressive realization* warrants caution and further study considering the impact of the cost-containment measures over time.

### Shared responsibility, shared decision making, attention to vulnerable and marginalized groups and non-discrimination

The last set of the indicators underly the legal principles of *shared responsibility, shared decision making, attention to vulnerable and marginalized groups* and *non-discrimination*. They give a much more ambiguous impression of its accordance to the right to health principles. The extent to which they are implemented according to a rights-based approach, and as such integrated in the Dutch health system, does not seem to be as readily apparent as the first proportion of indicators previously discussed. While adequate resources to deliver UHC are being mobilized, it remains to be seen whether this level of spending and redistribution of public revenue proves to be sustainable in the context of the demographic changes in the coming decades [36].

Furthermore, the Netherlands is a significant proponent of human rights, which it promotes in both domestic and international policy. However, the right to health as it is integrated in the Dutch international development agenda is specific to SRHR. The policy puts sexual and reproductive health, as well as gender perspectives and inequities, forward as a fundamental human rights issue. The Netherlands takes a prominent role within the international community in advocating for these rights [45, 47]. However, the health-related allocation of ODA on UHC or health system strengthening seem to be relatively insufficient compared to the spending on projects related to SRHR. This emphasis on SRHR that targets specific health services and programs might distract from investing in the more fundamental contributions to UHC and health system strengthening in low- and middle-income countries.

Lastly, the Netherlands does not implement an official SARA on the prioritization of marginalized groups. Asylum seekers are excluded from the Health Insurance Act and receive care through a parallel health system, with a central financial reimbursement policy for healthcare providers who provide services for undocumented migrants. While this policy ensures the availability of healthcare services for this group, it does not necessarily equate to adequate utilization of these services [59–61]. For example, the translation services have been defunded in an effort for cost-containment and to stimulate integration. This threatens the accessibility and acceptability of healthcare provision among patients with limited language proficiency (including but not limited to undocumented migrants) [62]. Therefore, a SARA should be implemented for undocumented migrants with special attention to the cultural access and gender and ethnic aspects. This is a vulnerable group that deserves careful attention in the context of UHC in HIC countries [25].

There is also no SARA implemented on participatory decision making in the Netherlands, neither in any of the other HIC’s [48]. A perspective of citizens in the decision-making process around the financing of health services from the collective funds is important yet limited. However, the recent non-governmental initiative *Burgerforum, keuzes in de zorg* aims to gather an insight into what society values concerning the financing of healthcare services from public funding [50]. This citizens panel is an attempt to address some important societal questions: which healthcare services are we willing to pay for each other and what do we value in making these choices? This deliberative panel is part of a larger societal debate on how resources for health can and should be distributed in the Netherlands [21, 41, 50, 63]. A health system co-owned by the population, communities and civil society is critical to strengthen health governance for UHC [64].

### Limitations

There are various comments and limitations to the design and findings of this review. A main limitation of a narrative literature review design is the less systematic and more iterative method of data searching. This can lead to a selection bias and subsequently influence the interpretation of the findings [27, 28]. While considered by the authors, the aim of the review is to present a broad and reflective scope of the topics at hand. Given time and resources, a narrative and iterative literature review design provided to be the most suitable. While this review is not exhaustive, it provides a concise and high-yield overview of UHC and the Right to Health in the context of the Netherlands. A first rapid review via several academic search engines indicated that PubMed and Google Scholar yielded qualitatively the most results. Other search engines like Web of Science and LexisNexis provided mainly duplication of results and few relevant additional data sources. Also, a rapid assessment of (legal) case studies provided little specific information that altered the overall analysis of this paper. Another limitation of this review relates to its focus on healthcare provision and the complexity of the Dutch healthcare system. Table 2  merely provides a brief overview of the main components of the health provision landscape. The way UHC is measured under the SDG framework is to a significant extent captured in the healthcare provision under the 2006 Health Insurance act, hence the focus of the review. The pressing availability of expensive treatment and medicines is also on the policy agenda and merits its own analysis [65, 66].

Also, the right to health requires greater attention to the realities of power dynamics, health and gender inequities and social determinants of health [67]. The Center for Economic and Social Rights addresses this issue through the OPERA framework; a tool designed to not only measure outcomes through indicators, but also by taking into account the policy efforts and commitments in monitoring the fulfillment of ECS rights. The OPERA framework has been developed to give a more comprehensive picture of compliance of states to the economic, cultural and social rights. It would be relevant to apply the OPERA framework to the Netherlands as to have a more complete image, from a specific health angle, of how it fulfills the ECS rights of its citizens.

As formulated in the ICESCR, the right to health mainly focuses on the equality of dignity, legal standing, and legal status, but lacks emphasis on the equality of social or economic positions. Inequalities in social class and economic status are hence, according the ICESR, of lesser significance, unless these interferes with the realization of the right to health. In other words, any inequalities above the minimum requirements to fulfilling the right to health are legally permitted. In addition, these underlying determinants of health tend to be considered individually and sequentially, and thus underestimate the interlinked and cumulative effects of the determinants [68]. While the right to health approach, as used this review, does address equity within the 10 indictors, a more thorough analysis of the concepts of *fairness* and *equity* of the health policy beyond the right to health is warranted. A path-dependency investigation of the observations made and an enquiry of the demands for health equity in relation to health care financing are interesting subjects for further studies.

### Policy and research implications

What should we expect from the Dutch government to be a decent way to move healthcare financing forward with an rights-based approach? Generating a sustainable and equitable financial revenue remains of uttermost importance for the future health policy agenda. There should be a progressive realization to the inclusion of healthcare services in the basic health package. It is important that these services, as well as the services already included, are evaluated for their cost-effectiveness as well as societal relevance. It therefore requires adequate participation of citizens in the decision-making process. Furthermore, the Netherlands needs to take more responsibility to the achievement of UHC in low-income countries if is to play its part in achieving the SDGs. This can be done by increasing the provision of financial means, via ODA, to projects earmarked, directly or indirectly, for UHC implementation. While these issues are important, the sustainability of UHC is not only a matter of economic or financial considerations. Attention should also be paid to the social and political dimensions, especially in the context of HIC’s. The increasing neoliberal approach to healthcare policy is argued to put pressure on the progressive realization of UHC in many European countries [63, 69]. When financial resources for healthcare are scarce, rooting healthcare provision in principles like the right to health might prevent a regressive policy [13, 24]. It is therefore of extra importance to not only adequately address the health of vulnerable people and marginalized groups in health policies, but also in the policy process itself by involving them and other citizens in the decision making around financing healthcare from public resources. One might say that the ability to protect and respect minority rights is one of the determinants of the decency of a democratic society [70].

Finally, the overall policy landscape in the Netherlands shows that UHC is based to a great extent on the right to health. Nonetheless, it raises questions on the underlying values of how the right to health is and could be interpreted, and how the current political climate frames healthcare. An enquiry of the right to health as a moral right would reflect on ethical questions considering legitimate, relevant or obligatory actions in healthcare. How ought healthcare (or even health itself) to be distributed? And how can this distribution be achieved in a fair and just manner? The right to health serves as a reference point for both ethical principles guiding our actions and a legal obligation with varying levels of enforcement [12]. The UHC debate in the Netherlands falls somewhat short of an ethical and philosophical enquiry into health system financing policies. This debate seems to be rather rational techno-economic in nature with an approach based on resource-oriented values [71]. Perhaps health equity and fairness are not as dominant of values in Dutch health policy as might be expected. Future research could also focus on unpacking the synergies and tensions between the economic and normative dimensions of health system financing. The right to health as a moral concept could be explored in the context of health system financing [13]. As policies on health system financing takes the direction of decentralizing and shifting to the private sector, the research agenda is seemingly directed towards the implementation of cost-containment measures. Deliberating the entirety of health system financing, for example by a complex system approach, is needed to tackle the most pressing healthcare issues the coming decades [72].

## Conclusion

The healthcare provision in the Netherlands is characterized by a techno-economic focus: all the essential services are covered, the mandatory nature of the insurance covers virtually the whole population, and pooled funding prevents catastrophic health expenditure. This approach towards achieving UHC is seen in the emphasis on the legal human rights principle of *minimum core obligations*. The *shared-responsibility, non-discrimination* and *participatory decision-making* aspects of public policy for UHC require more attention. However, the fiscal sustainability of healthcare is under pressure and the COVID-19 pandemic might exacerbate inequalities. A regressive healthcare policy might be prevented by basing it to principles and values of equity and fairness. Healthcare provision is a question of just distribution as much as cost-effectiveness.

## Data Availability

Data sharing is not applicable to this article as no datasets were generated or analyzed during the current study.

## Abbreviations

CESCR: Committee on Economic, Social and Cultural Rights
GDP: Gross Domestic Product
HIC: High-income country
ICESCR: International Covenant on Economic, Social and Cultural Rights
ODA: Official Development Aid
OECD: Organization for Economic Co-operation and Development
OOPP: Out-of-pocket payment
SARA: Service Availability and Readiness Assessment
SDG: Sustainable Development Goal
UHC: Universal Health Coverage
UN: United Nations
WHA: World Health Assembly
WHO: World Health Organization
